# Molecular characterization of T cell receptor beta variable in the peripheral blood T cell repertoire in subjects with active tuberculosis or latent tuberculosis infection

**DOI:** 10.1186/1471-2334-13-423

**Published:** 2013-09-08

**Authors:** Jiezuan Yang, Jianqin He, Haijun Huang, Zhongkang Ji, Li Wei, Ping Ye, Kaijin Xu, Lanjuan Li

**Affiliations:** 1 State Key Laboratory for Diagnosis and Treatment of Infectious Disease, the First Affiliated Hospital, College of Medicine, Zhejiang University, Hangzhou, China; 2 Collaborative Innovation Center for Diagnosis and Treatment of Infectious Diseases, Zhejiang University, Hangzhou, China; 3 Department of Infectious Disease, Zhejiang Provincial People’s Hospital, Hangzhou, China

## Abstract

**Background:**

T cells are closely linked to the clinical manifestations of subjects with *Mycobacterium tuberculosis* (MTB) infection. T cell receptor beta variable (TCRBV) is a signal and indicative molecule on the membrane of T lymphocytes, reflecting the composition and specificity of T cells. The molecular profiles of TCRBV in peripheral blood mononuclear cells (PBMCs) and their subpopulations (CD4^+^ and CD8^+^ T cells) from subjects with active tuberculosis (TB) or latent TB infection (LTBI) have not been well described.

**Methods:**

In 42 subjects with active TB or LTBI, PMBCs and their subsets were separated and sorted. The molecular profiles of the TCRBV complementarity determining region 3 (CDR3) in the three cell populations were investigated using our recently developed gene melting spectral pattern (GMSP) assay. The TCRBV members were then cloned and sequenced when their GMSP image profiles showed a single-peak.

**Results:**

The average number of skewed TCRBV molecules in the CD4^+^ cell subset was significantly higher than that in PBMCs and CD8^+^ T cells. TCRBV12, BV13.1, BV13.2, and BV24 were expressed more prevalently than other TCRBV gene families in the three cell populations. In addition, relatively conserved amino acid motifs were identified in TCRBV5.1 and BV20 CDR3 in PBMCs and its subsets. The monoclonal TCRBV14 and BV23 expressed were different between active TB and LTBI subjects.

**Conclusions:**

These results indicate that the T cell immune response is complex and multi-specific in active TB and LTBI subjects. Analysis of TCRBV expression in CD4^+^ T cells suggest that it could be useful in assessing the composition and status of circulating T cells. Furthermore, the expression of TCRBV14, BV23 and the sequencing of CDR3 amino acid motifs of TCRBV5.1, BV20 could be used in the differential diagnosis and treatment of subjects with active TB or LTBI.

## Background

Tuberculosis (TB) is the most common opportunistic infection caused by *Mycobacterium tuberculosis* (MTB), and is a serious global public health problem. According to the 2012 World Health Organization (WHO) report, about 1.4 million people, of which 500,000 were female, died from MTB infection. About 8.7 million subjects were newly infected with TB in 2011, including 500,000 children [1].

Clinical experience suggests that early diagnosis and treatment are key to improving the prognosis of TB patients, and are important factors in controlling the spread of TB [2], significantly reducing fatality. The commercial enzyme-linked immunospot assay (T-SPOT.TB) based on the interferon (IFN)-gamma release assay (IGRA) is useful in diagnosing subjects infected with MTB, with high sensitivity and specificity [3,4]. The T-SPOT.TB assay can help to distinguish subjects with latent TB infection (LTBI) from those vaccinated with Bacillus Calmette-Guerin (BCG) [5,6], but cannot directly distinguish between subjects with active TB and LTBI [7,8].

T cells play a key role in the control of MTB infection in *vivo*, especially the CD4^+^ T cell population [9-11]. The T cell receptor (TCR) repertoire can reflect the specificity, status, and composition of T cell populations. However, the molecular characterization of TCRBV in the peripheral blood of subjects with MTB infection is not well described [12]. Characterization of the TCR repertoire of T cells and its subsets (CD4^+^ and CD8^+^ T cells) in MTB subjects may help clarify the role of T cells in TB pathogenesis. Furthermore, understanding the difference in TCR expression between active TB and LTBI, may aid diagnosis and the development of personalized treatment in subjects with MTB, especially in active TB patients. In this study, CD4^+^ and CD8^+^ T cell subsets were obtained using a magnetic bead separation technique, and characterization of the TCRBV CDR3 in these cell populations as well as in peripheral blood mononuclear cells (PBMCs) from subjects with active TB or LTBI was investigated using gene melting spectral pattern (GMSP) assay.

## Methods

### Subjects

All participants were recruited in the First Affiliated Hospital, College of Medicine, Zhejiang University over a 6-month period from November 2011 through April 2012. A heparinized blood sample was drawn following informed consent. The study was conducted according to the guidelines of the Declaration of Helsinki. The medical ethics committee of the First Affiliated Hospital, Zhejiang University approved all procedures involving human subjects.

Forty-two subjects with a positive T-SPOT.TB assay were recruited. Of these, active TB was diagnosed in 22 cases including 15 with pulmonary tuberculosis, 4 with pulmonary tuberculosis with pleurisy, 2 with pulmonary tuberculosis and intestinal tuberculosis, and 1 case with tuberculous pleurisy. These 22 cases had positive sputum/bronchial alveolar lavage (BAL) acid fast bacilli (AFB) stains and/or culture for MTB. Sputum and BAL were cultured according to National Center for Clinical Laboratory Standards for 6 weeks in a liquid (BACTEC MGIT 960, BD Biosciences) and using a solid medium (BD Biosciences). The remaining 20 cases had no history of active tuberculosis, and the clinical diagnosis of latent tuberculosis infection (LTBI) was based on nucleic acid specimens tested positive by PCR for MTB and typical imaging information. This pattern with LTBI was more described in previously report [13]. Of these subjects, 1 also had irritable bowel syndrome (IBS), 3 had a small ulcer, 2 had pericardial effusion and 1 had pleurisy, and the remaining patients with latent pulmonary TB.

Subjects were excluded if they were co-infected with other infectious diseases, such as human immunodeficiency virus (HIV), or overt cytomegalovirus (CMV) infection. Subjects with severe liver disease and kidney failure were also excluded. None of the enrolled patients used immunosuppressive agents or enhancers. Additional information on the enrolled subjects is shown in Table 1.

**Table 1:** **Demographic and clinical characteristics of the subjects enrolled in the study**^**a**^

	**Total number**	**Active TB**	**LTBI**
	**(n = 42)**	**(n = 22)**	**(n = 20)**
Age	47.0 ± 14.1	46.2 ± 15.4	48.6 ± 13.1
Male	26	15	11
BCG			
Vaccinated	25	13	12
Unvaccinated	14	8	6
NA	3	1	2
T-SPOT. TB			
Positive	42	22	20
Negative	-	-	-

^a^All enrolled subjects are Chinese Han population.

*LTBI* latent *TB* infection, *TB* tuberculosis, *BCG* Bacillus Calmette - Guerin, *T-SPOT. TB* the commercial enzyme-linked immunospot assay, *NA* not available.

### Isolation of PBMCs, CD4^+^ and CD8^+^ T cell sorted

Peripheral blood mononuclear cells (PBMCs) from 42 subjects with active TB or LTBI were obtained from fresh heparinized blood by density gradient centrifugation on Ficoll-Paque (CEDARLANE, Netherlands). CD4^+^ and CD8^+^ T cells were positively selected from PBMCs using anti-CD4/anti-CD8 monoclonal antibodies coupled to antibody-coated magnetic beads (Miltenyi Biotec, Bergisch Gladbach, Germany), according to the manufacturer’s instructions. Purity of the separated CD4^+^ and CD8^+^ T cell populations were tested by Flow Cytometry (FCM) analysis using FITC-labeled anti-CD4 and PE-labeled anti-CD8 monoclonal antibodies, respectively. The T cell subpopulations were found to be more than 95% purity (data not shown).

### RNA extraction and cDNA synthesis

Total RNA was extracted from PBMCs (CD4^+^ and CD8^+^ T cell subpopulations) using a SV Total RNA Isolation System (Promega, Madison, WI, USA) according to the manufacturer’s instructions. RNA was quantified using a NanoDrop® ND-2000 spectrophotometer (Thermo Fisher Scientific, Wilmington, Delaware, USA), and its integrity was confirmed electrophoretically (including 28S and 18S band on 2% agarose gel electrophoresis). Total RNA was immediately reverse transcribed to cDNA using the RevertAid First Strand cDNA Synthesis Kit (MBI, Fermentas, EU) according to the manufacturer’s instructions. Briefly, 1–5 μg total RNA was reverse transcribed with OligodT_18_ as primer in a 20 μL reaction volume and stored at −25°C.

### GMSP assay of TCRBV in PBMCs, CD4^+^ and CD8^+^ T cells

The GoTaq® qPCR Master Mix (with Rox™ dye) (Promega, Madison, WI, USA) was used as the real-time PCR kit. A Mastermix of 25 μl for each of the 28 reactions containing 0.4 μM reverse primer TCRBC, and 50 ~ 150 ng template cDNA was prepared. For TCRBV gene families specific amplification, the corresponding forward primer (TCRBV1 ~ 24) was added to a final concentration of 0.4 μM. The real-time PCR reaction parameters were as follows: 2 min at 95°C to activate the GoTaq enzyme, followed by 45 cycles at 95°C for 15 s, 56.0°C for 25 s, and 72°C for 35 s with a final extension at 72°C for 8 min, following the PCR products melting curve analysis. The peak shape pattern of the melting curve for 24 TCRBV gene families was determined by plotting the first negative derivative of the decrease in fluorescence signal versus temperature (−dF/dT) versus temperature (Tm), generating gene melting spectral patterns (GMSPs) as previously reported [14,15]. The simultaneous amplification of TCR beta chain constant 1 (TCRBC1) and glyceraldehyde-3-phosphate dehydrogenase (GAPDH) gene segments were used as positive (monoclonal) controls.

### Relative frequency of TCRBV families

The expressing frequency of each TCRBV family in T-cell populations was calculated based on signal strength of the real-time PCR reactions, and expressed as the ratio of the copy number between TCRBV and GAPDH (R_BVx =_ 2^^Ct (GAPDH)-Ct (BVx)^), where Ct refers to threshold cycle. The relative TCRBVx gene frequency (%) was calculated according to the following formula [16,17].

$$\mathrm{TCRBVx}\left(\%\right)=\mathrm{RBVx}\times 100/\sum_{\times =1}^{24}\mathrm{RBVx}$$

### Identification of skewed TCRBV families

A skewed TCRB gene family was defined using the profile of each GMSP image displayed by the software (Opticon Monitor 3.0) attached to the MJ Opticon 2 DNA engine (Bio-Rad, USA), and included two categories: 1) “Oligoclonal expansion”, appearing as a main peak associated with other small peaks on the GMSP, and the small peak with a height less than half the height of the main peak; and 2) the “Monoclonal” category, with only one main peak, and a very short small peak or no additional peak. Further details are described in our previously published report [15].

### Cloning and sequencing

If a TCRBV gene family showed a monoclonal GMSP profile (single peak shape), the sample was selected for cloning and sequencing to determine the degree of homogeneity within the CDR3 region. Briefly, the PCR products were re-amplified using GoTaq DNA polymerase (Promega, Madison, WI, USA) by touchdown PCR. The parameters were as follows: pre-incubation at 95°C for 2 min, 95°C for 30 s, 60°C for 40 s, and 72°C for 45 s, for 8 cycles with annealing temperature decreasing 0.5°C per cycle, and 95°C for 45 s, 56°C for 45 s, and 72°C for 50 s, for 27 cycles. The cycling was followed by a terminal elongation step at 72°C for 8 min. The nest-PCR products were separated using 2% (0.5% Tris-buffered EDTA) agarose gels (FMV BioProducts, Rockland, ME, USA), excised, and purified using a QIAEX gel extraction kit (Qiagen, Germany). Purified PCR products were ligated into pGEMT-T using the pGEM-3Z Cloning Kit (Promega, Madison, WI, USA) according to the manufacturer’s instructions. Cloning details have been described previously [18]. The plasmid DNA was sequenced using an ABI3730 DNA Sequencer (Applied Biosystems, Foster City, CA, USA).

The resulting sequences (nucleotide) were compared against sequences in the IMGT-LIGM database (http://www.imgt.org), and translated into their corresponding amino acids using Chromas software version 2.22 (Technelysium Ltd., South Brisbane, Queensland, Australia). BLAST (http://blast.ncbi.nlm.nih.gov/Blast.cgi) was used to define CDR3 and BJ segments in the TCRBV gene families [17,19].

### Statistical analysis

All data were analysed using SPSS software version 16.0 (SPSS Inc., Chicago, IL, USA). The differences in skewed TCRBV gene families within PBMCs (CD4^+^ and CD8^+^ T cells) from subjects with active TB or LTBI were analysed using the Kruskal-Wallis H test or nonparametric Mann–Whitney *U* test. Differences in the data between two TCRBV families were examined using a *χ*^2^-test or Student’s *t*-test, with *p* < 0.05 considered statistically significant.

## Results

### Skewed TCRBV families within PBMCs, CD4^+^ and CD8^+^ populations

It is well known that the profile of TCRBV gene families in PBMCs from healthy donors often shows a multipeak-shaped pattern (Gaussian distribution) [14]. Moreover, no significant differences between vaccinated and unvaccinated subjects in both adult groups with active TB and LTBI were reported. In this study, the skewed TCRBV families were compared within the PBMCs, CD4^+^ and CD8^+^ T cell subsets isolated from subjects with active TB or LTBI. Of the three cell populations evaluated, the TCRBV families in the CD4^+^ T cell subset contained a greater number of skewed clonally expanded TCRBV families. Moreover, the average number of skewed (oligoclonal and monoclonal) TCRBV families in the CD4^+^ T cell population was higher than that in the PBMCs and the CD8^+^ T cell subset (*p* < 0.05, 0.01), and there was no significant difference between the latter two cell populations (Table 2).

**Table 2:** **The frequencies of skewed TCRBV in CD4**^+^, **CD8**^+ ^**T cells and PBMCs of the subjects with active TB or LTBI**^**a**^

**TCRBV families**	**CD4**^+ ^**T cells**	**CD8**^+ ^**T cells**	**Total PBMCs**
	**Incidence (%)**^**b**^	**Incidence (%)**^**b**^	**Incidence (%)**^**b**^
1	8	5	7
2	9	8	7
3	14 (35.0)	8	11 (28.2)
4	7	5	7
5.1	15 (37.5)	9	12 (30.8)
5.2	7	9	8
6	8	5	6
7	7	6	4
8	10 (25.0)	7	6
9	7	5	3
10	7	6	8
11	8	3	7
12	24 (60.0)	16 (41.0)	21 (53.8)
13.1	16 (40.0)	12 (30.8)	12 (30.8)
13.2	15 (37.5)	13 (33.3)	15 (38.5)
14	11 (27.5)	8	8
15	9	5	7
16	7	5	2
17	6	6	5
18	10 (25.0)	9	8
19	7	7	5
20	14 (35.0)	8	11 (28.2)
21	7	5	6
22	7	6	5
23	10 (25.0)	7	8
24	16 (40.0)	10 (25.6)	12 (30.8)
Total no. of skewed BV (average ratio for a case)	266 (6.65) ^c^	193 (4.95) ^c, d^	211 (5.41) ^c, d^
No. of subjects examined with normal pattern (ratio, %)	2 (4.76) ^e^	3 (7.14) ^e^	3 (7.14)^e^
No. of subjects examined	42	42	42

^a^The number of TCRBV gene families showing skewed-clone expansion (oligoclonal or monoclonal) is summarized in subjects with active TB or LTBI.

^b^The number of samples showing a skewed-clone expansion in total detected samples (percentages of each TCRBV skewed-clone expansion). Sample with normal GMSP (no skewed-clone expansion pattern in any TCRBV families in a subject) are excluded in the percentage calculation, and the greater than or equal 25% percentages are shown only.

^c^The average expansion rate of TCRBV gene families was higher in CD4^+^ T cell subset than in other two cell populations (*P* < 0.01, 0.05 by *χ*2 test).

^d^There was no significant difference between the two groups (*P* > 0.05 by *χ*^*2*^ test). Sample with normal GMSP (no biased-clone expansion pattern) are excluded in the average ratio calculation.

^e^The incidence of the normal GMSP was significantly lower in CD4^+^ T cells than in other two cell populations (*P* < 0.01 by *χ*^*2*^ test).

In addition, there were fewer subjects with a normal TCRBV pattern in the CD4^+^ T cell population compared with the two other cell populations, and four TCRBV gene families (BV12, BV13.1, BV13.2, and BV24) were more prevalent than other TCRBV members in the three cell populations derived from subjects with active TB or LTBI (Table 2).

### Skewed TCRBV profiles within CD4^+^ and CD8^+^ populations

**Figure 1:**
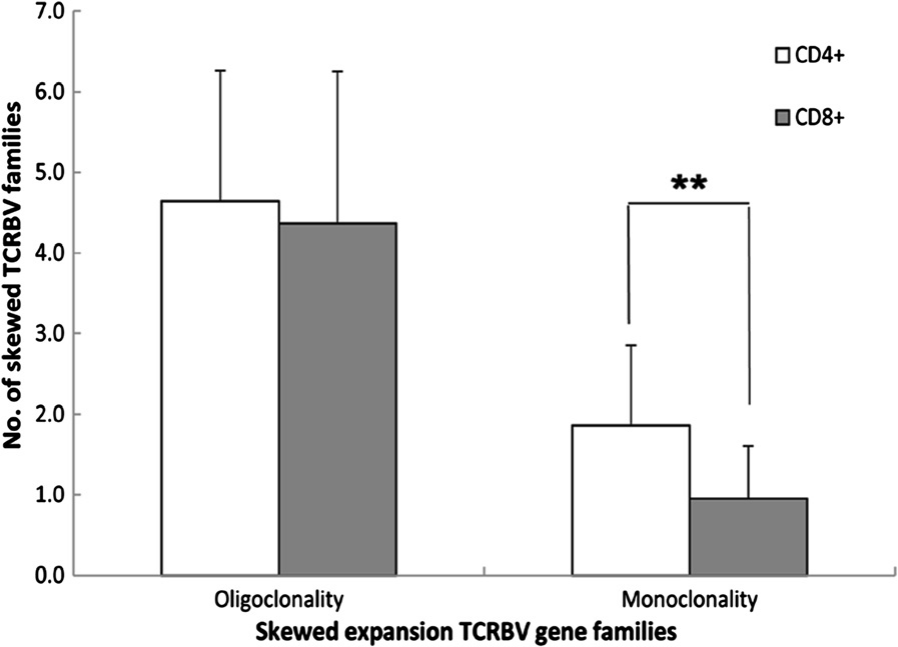
**Comparing the number of skewed TCRBV family between CD4**^+ ^**and CD8**^+ ^**T cell subsets in active TB patients.** Values are means ± STD. **P <0.01. CD4^+^ cell, purified CD4^+^ T lymphocytes; CD8^+^ cell, purified CD8^+^ T lymphocytes.

**Figure 2:**
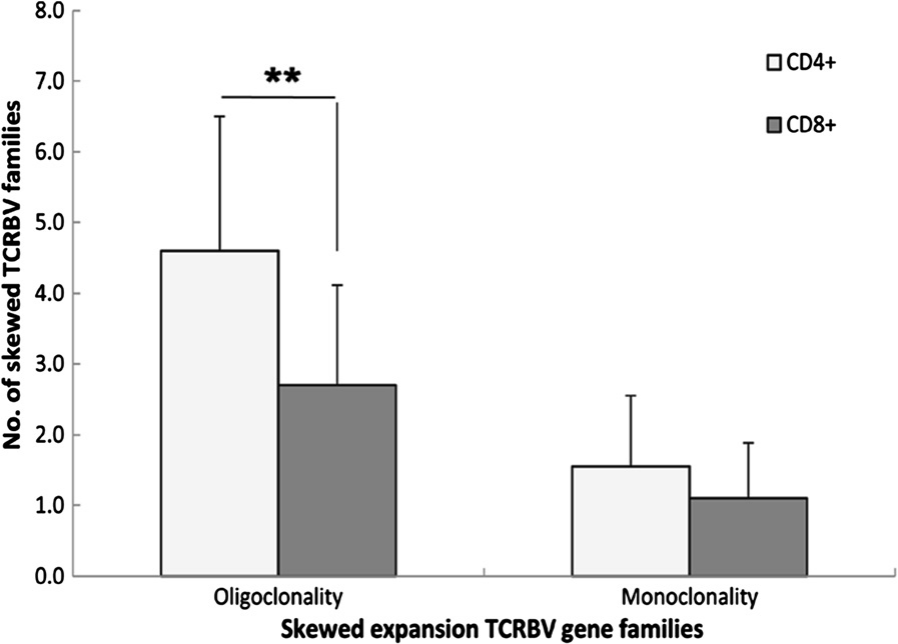
**Comparing the number of skewed TCRBV family between CD4**^+ ^**and CD8**^+ ^**T cell subsets in LTBI individuals.** Values are means ± STD. **P <0.01. CD4^+^ cell, purified CD4^+^ T lymphocytes; CD8^+^ cell, purified CD8^+^ T lymphocytes.

A greater number of skewed TCRBV families was expressed in the CD8^+^ T cell population from patients with active TB than from those with LTBI (*p* < 0.05), and there was no significant difference in skewed TCRBV families in the CD4^+^ T cell population between subjects with active TB or those with LTBI (*p* > 0.05) (Table 3). To further distinguish the skewed expansion features of the TCRBV family in active TB and LTBI subjects, the profile of oligoclonal and monoclonal expansion of TCRBV families from CD4^+^ and CD8^+^ T cells was analysed. We found that there was a higher number of monoclonal expansion TCRBV patterns in the CD4^+^ fraction compared with the CD8^+^ fraction in active TB patients (Figure 1). Regarding LTBI subjects, there was also a higher number of oligoclonal TCRBV patterns in the CD4^+^ T cell subset (Figure 2).

**Table 3:** **Average ratio of skewed TCRBV gene families in CD4**^+ ^**and CD8**^+ ^**T cell subsets of subjects with active TB or LTBI**

	**Active TB**		**LTBI**	
	**CD4**^+ ^**T cells**	**CD8**^+ ^**T cells**	**CD4**^+ ^**T cells**	**CD8**^+ ^**T cells**
Total no. of skewed TCRBV	143	117	123	76
Total no. of subjects detected	22	22	20	20
No. of subjects examined with normal pattern	1	1	1	2
Average ratio of skewed TCRBV (no. of subjects with skewed TCRBV)	6.81 (21)^a^	5.57 (21)^b^	6.47 (19)^a^	4.22 (18)^b^

^a^The rate of skewed TCRBV family was not significantly different between the two groups (*P* > 0.05 by *χ*2 test).

^b^The rate of skewed TCRBV family was higher in active TB group than that in LTBI group (*P* < 0.05 by *χ*2 test).

### Relative frequency of each TCRBV family in PBMCs

**Figure 3:**
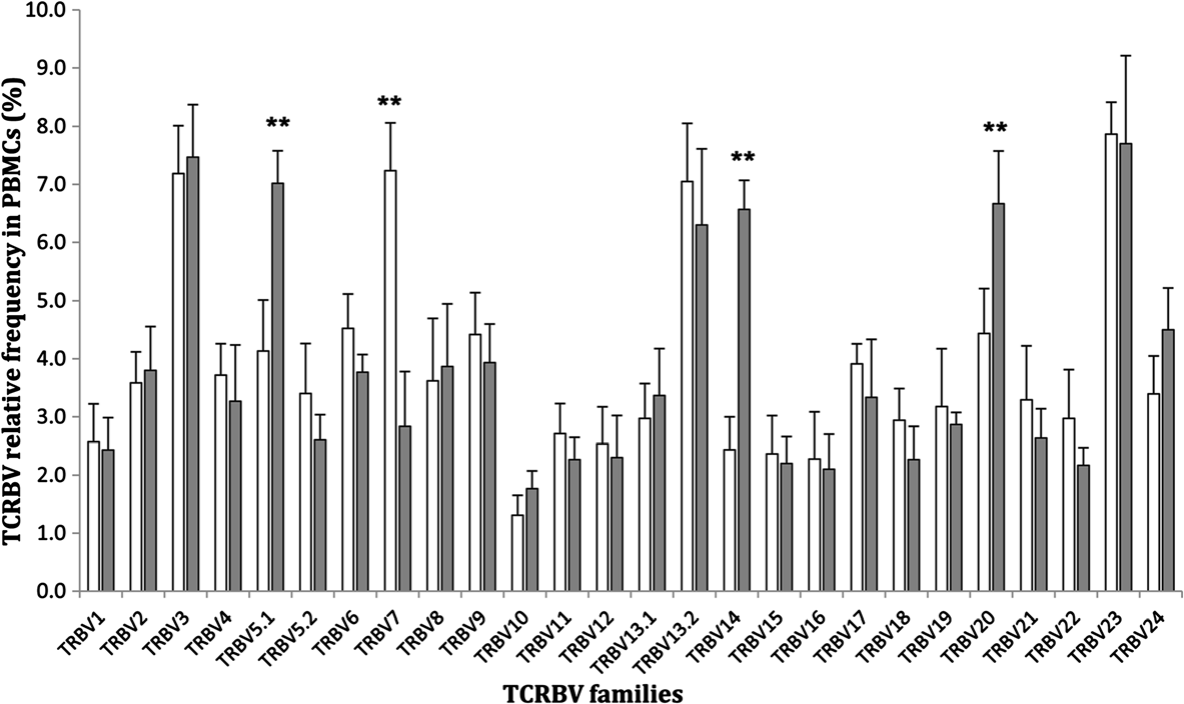
**Relative frequency of each TCRBV family in active TB or LTBI subjects.** TCRBV family in PBMCs derived from active TB patients or LTBI individuals were shown as open bars or filled bars respectively. Data are presented as % expression of each TCRBV family relative to the total expression of all 24 TCRBV genes (1 ~ 24). The data represent the means and standard deviation. **P <0.01.

The relative percentage (%) of a TCRBV family determined the relative frequency in PBMCs derived from active TB and LTBI subjects. We observed that the frequency of most TCRBV families was not significantly different between the two groups. Furthermore, we found that the frequency of TCRBV families (BV7) in the LTBI group was lower than that in the active TB group (*p* < 0.01). However, the former group had three TCRBV families (BV5.1/BV14/BV20) with higher expression than that of the latter group (*p* < 0.01) (Figure 3).

### TCRBV CDR3 motifs in PBMCs, CD4^+^ and CD8^+^ populations

When the profile of a TCRBV gene family expressed a monopeak-shaped pattern, the TCRBV family was cloned, sequenced and translated to the corresponding amino acid sequence. Representative amino acid sequences of TCRBV CDR3 in PBMCs, CD4^+^ and CD8^+^ T cell populations from active TB and LTBI subjects are shown in Tables 4 and 5, respectively.

**Table 4:** **Representative amino acid sequences of monoclonal TCRBV families within PBMCs and CD4**^+^, **CD8**^+ ^**T cell populations of active TB patients**^**a**^

**Patients**^**b**^	**Vbeta**		**CDR3**	**BJ**		**Ratio**^**c**^
J1 (CD4+)	BV24	AAMYLCATS	SDRVSPL	HFGNGTRLTVTED	1.6	21/25
J16 (CD4+)	BV24	AAMYLCATS	QDRAKQ	YFGPGTRLLVLED	2.1	20/23
J5	BV23	SALYFCASS	NTGTSDGY	TFGSGTRLTVVED	1.2	21/25
J2 (CD4+)	BV23	SALYFCASS	NTGTSDGY	TFGSGTRLTVVED	1.2	20/23
J2	BV20	SGFYLCAWS	TGTGHSPL	HFGNGTRLTVTED	1.6	25/26
J5	BV20	SGFYLCAWS	TGTGHSPL	HFGNGTRLTVTED	1.6	23/26
J5 (CD4+)	BV20	SGFYLCAWS	TGTGHSPL	HFGNGTRLTVTED	1.6	21/26
J15	BV13.2	TSVYFCASS	YGPSTGEL	FFGEGSRLTVVED	2.2	20/23
J11 (CD8+)	BV13.2	TSVYFCASS	YSTDEQ	YFGPGTRLTVTED	2.7	18/26
J22	BV13.1	TSVYFCASS	YWGSDTQ	YFGPGTRLTVLED	2.3	17/25
J13	BV12	TSVYFCAIR	KQGDSYEQ	YFGPGTRLTVTED	2.7	14/22
J17 (CD8+)	BV12	TSVYFCAIR	KQGDSYEQ	YFGPGTRLTVTED	2.7	18/24
J20 (CD4+)	BV12	TSVYFCAIR	KQGDSYEQ	YFGPGTRLTVTED	2.7	17/22
J5	BV5.1	SALYLCASS	LDYSGNTI	YFGEGSWLTVVED	1.3	20/26
J1 (CD4+)	BV5.1	SALYLCASS	LDYSGNTI	YFGEGSWLTVVED	1.3	21/25
J3 (CD8+)	BV5.1	SALYLCASS	LDYSGNTI	YFGEGSWLTVVED	1.3	19/21
J10	BV3	SMYLCASS	LGARLSNQPQ	HFGDGTRLSILED	1.5	16/21
J4 (CD8+)	BV3	SSNFCSTT	LIGRAGATRA	YFGPGTRLTVTED	2.7	20/25

^a^When the GMSP profile of a TCRBV family displayed a single peak, the corresponding PCR products re-amplified, and the CDR3 were sequenced after cloning.

^b^The T cell subsets (CD4^+^ or CD8^+^) for analysis are indicated in brackets.

^c^The amino acid sequences of the max rate of TCRBV families are shown.

**Table 5:** **Representative amino acid sequences of monoclonal TCRBV families within PBMCs and CD4**^+^, **CD8**^+ ^**T cell populations of LTBI subjects**^**a**^

**Subjects**^**b**^	**Vbeta**		**CDR3**	**BJ**		**Ratio**^**c**^
J24 (CD4+)	BV24	AAMYLCATS	SDRVSPL	HFGNGTRLTVTED	1.6	22/25
J23	BV20	SGFYLCAWS	VQVGWGETQ	YFGPGTRLLVLED	2.5	12/21
J24 (CD4+)	BV20	SGFYLCAWS	TGTGHSPL	HFGNGTRLTVTED	1.6	21/25
J28 (CD8+)	BV20	SGFYLCAWS	TGTGHSPL	HFGNGTRLTVTED	1.6	20/24
J38	BV20	SGFYLCAWS	TGTGHSPL	HFGNGTRLTVTED	1.6	22/25
J32 (CD4+)	BV14	SLYFCASS	PGTTQETQ	YFGSGTRLLVLED	2.5	18/23
J25	BV14	SLYFCASS	PGTTQETQ	YFGPGTRLTVLED	2.5	15/23
J28 (CD8+)	BV14	SLYFCASS	RHTGGTEA	FFGQGTRLTVVED	1.1	19/30
J29	BV13.2	TSVYFCASS	YDENEQ	FFGPGTRLTVLED	2.1	16/22
J31 (CD4+)	BV13.2	TSVYFCASK	TKPVGEQ	FFGPGTRLTVLED	2.1	15/23
J42	BV13.1	TSVYFCASS	YSAGGPNEQ	FFGPGTRLTVLED	2.1	18/25
J34 (CD4+)	BV12	TSVYFCAIR	KQGDSYEQ	YFGPGTRLTVTED	2.7	17/23
J35	BV12	TSVYFCASR	VRLITNYGY	TFGSGTRLTVVED	1.2	24/24
J39 (CD4+)	BV12	TSVYFCATA	GGAPGQPQ	HFGDGTRLSILED	1.5	16/24
J23	BJ5.1	SALYLCASS	LDYSGNTI	YFGEGSWLTVVED	1.3	22/27
J25 (CD8+)	BJ5.1	SALYLCASS	LDYSGNTI	YFGEGSWLTVVED	1.3	22/27
J30	BJ5.1	SALYLCASS	LDYSGNTI	YFGEGSWLTVVED	1.3	22/25
J24	BV3	SMYLCASG	PNYYEQ	YFGPGTRLTVTED	2.7	23/25

^a^When the GMSP profile of a TCRBV family displayed a single peak, the corresponding PCR products re-amplified, and the CDR3 were sequenced after cloning.

^b^The T cell subsets (CD4^+^ or CD8^+^) for analysis are indicated in brackets.

^c^The amino acid sequences of the max rate of TCRBV families are shown.

Of the three cell populations from active TB and LTBI subjects, four TCRBV gene families (BV12, BV13.1, BV13.2, and BV24) were more prevalent than others. The amino acid sequence of some mono-clone expansion BV24 CDR3s were expressed as “SDRVSPL” with BJ1.6 or “QDRAKQ” with BJ2.1, and BV12 CDR3 were found expressed as “KQGDSYEQ” with BJ2.7 in active TB patients. Additionally, there were no identical amino acid sequences found in TCRBV13.1 and BV13.2 gene families in PBMCs, CD4^+^ and CD8^+^ T cell populations from active TB or LTBI subjects.

In addition, TCRBV5.1 and BV20, which were mainly derived from PBMCs or CD4^+^ T cell subsets in active TB and LTBI subjects, were predominant among all TCRBV families. Furthermore, the majority of CDR3 amino acid sequences of TCRBV5.1 and BV20 were expressed as “LDYSGNTI” with BJ1.3 and “TGTGHSPL” with BJ1.6, respectively. Similarly, the TCRBV3 family, with the majority found in PBMCs or CD4^+^ T cell subsets, was more prevalent than other TCRBV families, but there were no identical amino acid sequences found in this family.

However, among all the skewed TCRBV14 families in PBMCs, CD4^+^ and CD8^+^ T cell populations from active TB patients, there were no mono-clone TCRBV gene families detected. Similarly, there were no mono-clone TCRBV gene families detected among skewed TCRBV23 in LTBI subjects.

## Discussion

Tuberculosis (TB), a major cause of global mortality, has become increasingly prevalent and deadly due to the emergence of extensive drug resistant strains of MTB and the HIV/AIDS pandemic [20]. It has been reported that CD4^+^ T cell-mediated immunity plays a critical role in controlling chronic bacterial and viral infections [10,21-24]. In the present study, we showed that the use of our gene melting spectral pattern (GMSP) assay to study TCRBV gene families in PBMCs, CD4^+^ and CD8^+^ T cell subsets from subjects with active TB or LTBI was helpful in the differential diagnosis and treatment of active TB or LTBI. However, this will need to be further confirmed in a larger cohort.

T cells are a core component of adoptive immunity. They are divided mainly into two subsets, CD4^+^ and CD8^+^ T cells, and there are characteristic TCR molecular chains expressed on their respective cell membranes [25,26]. In the peripheral blood of healthy donors, the TCR in more than 95% of T cells is composed of alpha and beta chain heterodimers. Each T cell clone expresses a unique TCR that recognizes antigen-derived peptides bound to the major histocompatibility complex (MHC). The TCR has three complementary determining regions (CDR1, CDR2 and CDR3). CDR3 is the key determinant of T cell antigen specificity and mediates T cell diversity [27,28].

Analysis of the TCRBV repertoire has been widely used to characterize alterations in T cell repertoires in the peripheral blood of subjects infected with bacteria or viruses [29,30]. For instance, Wang *et al*. reported that a highly diverse TCR repertoire may be an important benchmark and target in the success of immune treat human CMV infection [31]. Vigano *et al*. described that the increased TCR renewal may provide the mechanistic basis for the generation of high-avidity HIV-specific CD8 T cells [32]. That the initial antiviral response in humans is maintained for many years in latent virus infected individuals was reported by Klarenbeek *et al*. [33]. In addition, Naumov et al. examined the TCR repertoire of CD8 T cells reactive against the influenza A viral epitope M1 (58–66) [34]. These studies were carried out usually using the high throughput sequencing (HTS), flow cytometry or molecular cloning techniques. In a previous study, we developed a GMSP assay to determine the clonal expansion status of TCRBV genes in PBMCs, and found that TCRBV11 may be associated with HBV replication in patients with chronic hepatitis B [15,35]. In the present study, the GMSP assay was used to determine the molecular property of skewed TCRBV gene families in PBMCs and CD4^+^ and CD8^+^ T cell subsets from active TB and LTBI subjects.

We directly analysed the degree of clonally expanded skewed TCRBV families in PBMCs, CD4^+^ and CD8^+^ T cell subpopulations from subjects with active TB or LTBI. We found that the average number of skewed TCRBV families for the three cell populations was 5.41, 6.65 and 4.95, respectively. Also, the average number of skewed TCRBV genes in the CD4^+^ T cell subset was significantly higher than that in the other two cell populations. This indicates that more CD4^+^ T cells contribute to the immune response in active TB or LTBI subjects than the other two cell populations. In addition, analysis of the TCR variable gene repertoire in blood or tissues may provide important information about the immune response against pathogens or immunopathological mechanisms [30,36]. Moreover, it has been reported that multispecific CD4^+^ T cells correlate with the control of MTB infections [9,12,37]. This suggests that the prevalent CD4^+^ T cells may be associated with the kind of CD4^+^ T cells controlling MTB in active TB or LTBI subjects, although, this will need to be determined in more cases.

In addition, we found that in patients with active TB, a higher number of monoclonal expansions were detected in CD4^+^ T cells compared with the CD8^+^ T cell subset. In LTBI subjects, the higher number of oligoclonal expansions was found in the CD4^+^ T subset too. This further suggests that CD4^+^ T cells may be associated with the host immune response to MTB infection and the different clinical manifestations between active TB and LTBI [38,39].

The T-SPOT.TB assay has been used to diagnose subjects with active TB or LTBI with high specificity and sensitivity. However, the assay does not permit distinction of active TB from LTBI [7,40], which has been tried by many researchers. Sester, *et al*. attempted to distinguish active TB from non-active states by the analysis of antigen-specific CD4^+^ T-cell cytokine profiles in peripheral blood [41], and Nemeth, *et al*. also want to discriminate between active TB and other diseases using cytokine profile [42]. Delogu, *et al*. reported that the T cell response to a recombinant and methylated heparin-binding haemagglutinin (HBHA) of MTB produced in *M*. *smegmatis* was useful in discriminating between active and non-active TB disease [43]. In this study, we found that the monoclonal expansion of TCRBV23 was only detected in the peripheral blood of patients with active TB, and TCRBV14 was only detected in LTBI, which may help distinguish patients with active TB from those with LTBI, although further studies are needed, with a larger sample size to improve accuracy of the method. In addition, between active TB and LTBI, the average number of skewed TCRBV in the CD4^+^ T cell subset did not differ significantly, but in the CD8^+^ T cell subset, there was a statistically significant difference.

Recent studies have reported on the development of TCR gene-modified T cells that allow the rapid generation of large numbers of cells with antigen-specificity and functional avidity, with the potential for clinical application [44-48]. The results of Luo showed that *M*. *tuberculosis* 38-kDa antigen-specific HLA class I and class II-restricted TCR genes can be successfully cloned and transduced into primary CD4^+^ and CD8^+^ T cells which result in T cells with stronger anti-MTB activity [49]. Thus, characterization of the MTB-specific T cells, especially their TCR gene usage [50], is essential for elucidation of the pathogenesis of active TB or LTBI and for the development of individualized treatment.

In the present study, we found that the skewed populations expressing the TCRBV12/BV13.1/BV13.2 or BV24 molecule were most prevalent compared with cell populations expressing other TCRBV gene families, regardless of the CDR3 in PBMCs, CD4^+^ or CD8^+^ subsets from active TB patients or LTBI individuals. Additionally, the frequency of three TCRBV families (BV5.1/BV14/BV20) in T cell populations in the LTBI group was higher than that in the active TB group. It was reported that the repertoire of TCRBV families present in patients with active TB might be considered defective in controlling the pathogen, since these patients are not able to control their infection. Whereas those patients with LTBI have a TCRBV repertoire that shows differences in public or private TCR families, rendering them capable of controlling the growth of the pathogen [11,51]. This indicates that high frequency TCRBV families (BV5.1/BV14/BV20) may be associated with immune control of the growth of MTB in LTBI subjects.

We further analysed CDR3 amino acid sequences in the monoclonal expansion TCRBV gene families, and found that there were no identical sequences of CDR3 among the four TCRBV gene families, which indicates that different T cell clones are involved in the immune response to MTB. The reasons for this may include the following: (1) there are different HLA phenotypes among different MTB subjects, which lead to different epitopes in MTB-specific immune T-cells, resulting in the proliferation of corresponding T cell clones; (2) there are various and scattered antigenic epitopes in MTB that are related to their pathogenicity and immunity; (3) the different sequences of CDR3 with similar spatial structure are likely to recognize the same epitope. However, we found that two CDR3 amino acid sequences had a relatively conserved motif (BV5.1, “LDYSGNTI”; BV20, “TGTGHSPL”) in PBMCs and CD4^+^ T cell populations from subjects with MTB infection, although, at present, it is not clear if or how the emergence of the relatively conserved TCRBV gene families influence the course of active or latent MTB infection and their prognoses.

## Conclusions

In the present study, we showed that the role of T cells in the immune pathogenesis of active TB or LTBI was complex and multi-specific. Analysis of TCRBV expression in CD4^+^ T cells was more useful in assessing the status and specificity of circulating T cells in subjects with active TB or in those with LTBI. The expression of TCRBV14, BV23 and the sequencing of CDR3 amino acid motifs of TCRBV5.1, BV20 could help in the differential diagnosis and treatment of active TB or LTBI, although, this should be confirmed in more cases, and with non-TB being excluded from LTBI subjects [11].

## Abbreviations

CDR3: Complementarity determining region 3; GMSP: Gene melting spectral pattern; IGRA: IFN-gamma release assay; LTBI: Latent TB infection; MTB: *Mycobacterium tuberculosis*; TB: Tuberculosis; TCRBV: T cell receptor beta variable.

## Competing interests

The authors declare that they have no competing interests.

## Authors’ contributions

JZ contributed to the study design, data collection, most experiments, and the writing the initial draft and revising the manuscripts. JQ and HJ collected the preliminary data, and helped to perform some experiments. ZK and WL participated in the study design and interpretation of the data. YP and KJ assisted in experimental design and help to data collection. LJ contributed to the study coordination, technical issues and revision of the manuscript. All authors read and approved the final manuscript.

## Pre-publication history

The pre-publication history for this paper can be accessed here:

http://www.biomedcentral.com/1471-2334/13/423/prepub
